# The Acceptability of Physical Activity to Older Adults Living in Lower Socioeconomic Status Areas: A Multi-Perspective Study

**DOI:** 10.3390/ijerph182211784

**Published:** 2021-11-10

**Authors:** Angela Devereux-Fitzgerald, Rachael Powell, David P. French

**Affiliations:** 1 Division of Nursing, Midwifery and Social Work, School of Health Sciences, University of Manchester, Manchester M13 9PL, UK; 2 Manchester Centre for Health Psychology, Division of Psychology & Mental Health, School of Health Sciences, University of Manchester, Manchester M13 9PL, UK; rachael.powell@manchester.ac.uk (R.P.); David.french@manchester.ac.uk (D.P.F.)

**Keywords:** inequality, ageing, deprivation, physical activity, exercise, acceptability, trainers, providers, qualitative

## Abstract

Older adults in lower socioeconomic status (SES) areas are the least active of all adult groups but are often absent from physical activity research. The present study aimed to elicit perspectives on acceptability of physical activity from older adults and physical activity providers in lower SES areas. Semi-structured interviews were conducted with 19 older adults and eight physical activity trainers/providers in lower SES areas. An inductive, multi-perspective Thematic Analysis was conducted. Eight themes were identified that covered one or both groups’ perceptions of what was important in ensuring acceptability of activity provision. Older adults perceived a lack of value that was reinforced by lack of resources and unequal provision. Acceptability was hindered by centralisation of facilities and lack of understanding of needs by facility management. Facilitating social interaction within physical activities appeared key, thereby meeting multiple needs with fewer resources. In conclusion, to increase acceptability of physical activity for older adults in low SES areas, providers should address the lack of perceived value felt by many older adults. Equitable provision of physical activities addressing multiple needs may allow older adults with limited resources to be physically active without sacrificing other needs. Facilitating creation of social bonds may foster maintenance of physical activities.

## 1. Introduction

Physical activity provides multiple benefits for older adults, including lowered risk of chronic illness and mortality, maintenance of cognitive and physical function, improved mood, and increased quality of life [1,2]. However, physical activity declines with age [3], with the majority of older adults (65+ years) in England not meeting current physical activity guidelines [4]. People in the most deprived areas are twice as likely to be inactive as those in the least deprived areas [5]. Furthermore, older adults in lower socioeconomic status (SES) areas can experience greater environmental and individual barriers to engaging in leisure-time physical activity than the general older adult population [6,7,8,9]. Despite these factors, older adults in lower SES areas are often absent from qualitative studies concerning both the concept of physical activity and engagement with behaviour-change interventions to increase physical activity [10,11].

If the acceptability of behaviour change interventions is overlooked, their effectiveness may be undermined [12]. Acceptability of health behaviour interventions has generally been conceptualised as the level of tolerance required to undertake health interventions or, more recently, the perceived appropriateness to those delivering or receiving a healthcare intervention based on their cognitive and emotional responses [13]. However, physical activity differs from many health behaviours, such as screening and adherence to medication, as it may be viewed as a desirable activity of itself—a pleasurable way to spend time connecting with others or reconnecting with oneself [14]. Acceptability of physical activity is therefore a more nuanced concept, incorporating context, such as resources, setting, delivery, experience, and meaning of physical activity, in order to determine individual acceptability [10,11,15]. Hence, simply identifying older adults’ motivations, beliefs, and barriers to engaging in physical activity (e.g., [16]) may pay insufficient attention to the impact of context.

Those who provide or deliver physical activity (hereafter referred to as trainers/providers) are key aspects of this context. King [8] noted that interpersonal approaches are most effective for engaging older adults in physical activity, with an incremental, empathic approach to delivery being highly acceptable [11]. It has been suggested that perceptions of individual characteristics of trainers/providers (including attitude, age, experience, training) influence engagement at the start of a physical activity programme as well as maintenance of physical activity in the general older adult population [17,18]. However, there has been little qualitative research into the experiences of those providing physical activity to older adults in low SES areas, where greater barriers exist. For example, two recent systematic reviews of the experiences of older adults regarding physical activity found only 1 out of 17 studies included low SES adults [10,11].

To improve services and increase engagement, we need to better understand what constitutes acceptable physical activity provision for older adults. We also need to understand what those who provide and deliver physical activity services within low SES locations perceive to be acceptable to older adults. The present qualitative study therefore aimed to elicit and analyse the views of older adults living in lower SES areas around the acceptability of engaging in physical activity to better inform future provision of physical activity services within these areas. It also aimed to elicit and analyse what trainers/providers perceive to be important in ensuring acceptability of activity provision for older adults in lower SES areas. Gaining the perspectives of both groups allows different but related issues to be examined concurrently, giving greater insight to the overall phenomenon as well as triangulating the data [19].

## 2. Materials and Methods

### 2.1. Design

A multi-perspective design used semi-structured interviews to elicit views and experiences of older adults and trainers/providers in relation to the acceptability of physical activity services to older adults.

### 2.2. Participants

All participants were recruited from lower SES areas of Manchester, England, a city which itself is deprived relative to much of the rest of the country. Fifteen areas within the city were selected based specifically on the percentage level of deprivation that older adults in those areas experienced [20] in order to target participants living and working in the most relevant context for the study. In the eligible areas at the time of recruitment, between 38.5 per cent and 54.8 per cent of older adults were living in deprivation (compared to the national English average of 18.1 per cent), where deprivation was based on factors including crime risk, living environment, access to local amenities, and income [20].

Older adults were eligible to take part if they were aged at least 65 years, lived independently in the lower SES areas described, had sufficient English language capability for an interview, and could walk without stopping for 10 min without assistance (walking aids permissible). Trainers/providers were eligible if they were over 18 and involved in delivering physical activity classes or services to adults 65 years old and over in a paid or voluntary capacity and in any of the specified lower SES areas. Purposive sampling aimed for variation in the older adult sample in terms of activity levels, age, and residential area, and for trainers/ providers, type of sector serviced, and type of activity provided. Service area data collection took place March–July 2015. Interviews were conducted until data saturation was reached. Initial recruitment was publicised via age-related charities, newsletters (research groups and local government), and libraries, together with a snowballing approach where further possible participants were identified by those who had already taken part. The first author visited coffee mornings, community social and craft groups, and physical activity sessions aimed at older adults to enhance recruitment, as face-to-face recruitment regarding physical activity research has been found effective in the older adult population [21]. The first author was familiar with similar low SES settings and seemed readily accepted by participants.

### 2.3. Procedure

Institutional ethical approval was granted. Eligibility was established and participant information sheets delivered. Informed consent (including for use of anonymized quotes) was gained prior to face-to-face interviews, which were conducted by the first author at the participant’s home or work or at the first author’s university. The interviews consisted of two parts: a structured questionnaire to obtain demographic and background data, immediately followed by the semi-structured interview. No financial remuneration was provided beyond travel expenses for those who attended the university. Data from previous interviews were considered throughout in an iterative process, so emerging topics could be addressed in later interviews. This process continued until data saturation became apparent. Field notes were taken after interviews to retain context. Interviews lasted 31–95 min (median 61 min) and were audio-recorded.

### 2.4. Materials

The structured questionnaires covered demographic questions and included items about physical activity levels and car ownership (older adults) and work role, delivery sector, and physical activity type provided (trainers/providers). Older adults’ physical activity levels were established based on the amount of time (min per week) participants self-reported spending on light, moderate, or vigorous physical activities: they were denoted as highly active if they met the recommended guideline of 150 min of moderate/75 min of vigorous physical activity per week plus worked on strength building and flexibility [2], active if they met the 150 min of moderate/75 min of vigorous physical activity per week and did not report engaging in strength/flexibility activities, somewhat active if they did some moderate/vigorous physical activity per week but did not meet the guidelines, and lower activity if they did not report engaging in any physical activity beyond basic daily living.

Semi-structured interviews were facilitated by interview schedules focusing on factors of acceptability of physical activity, updated as necessary with suggested topics from ongoing interviews with both groups. The older adults’ interview schedule (see Supplementary S1) included how participants felt about physical activity, their physical activity levels, physical activity likes/dislikes, benefits or concerns, and local physical activity provision. The trainers/providers’ interview schedule (see Supplementary S2) included motivation for working with older adults and physical activity, what older adults wanted regarding physical activity, local physical activity provision, barriers for older adults, attendance and feedback received, and experiences of increasing older adults’ physical activity engagement.

### 2.5. Analysis

**Figure 1:**
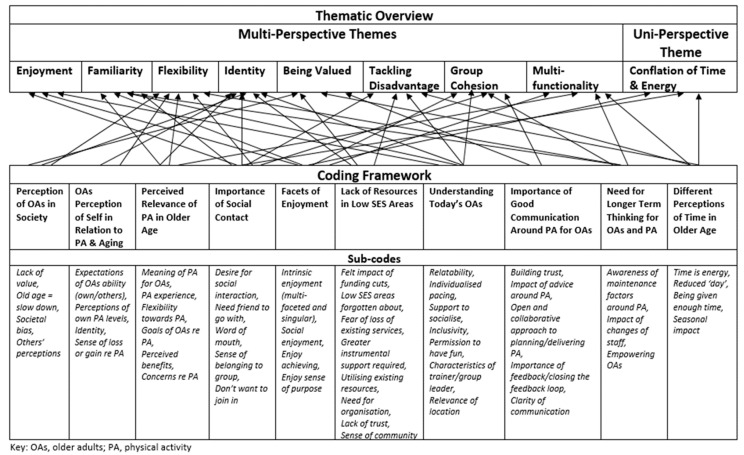
Thematic overview showing relationship between themes and coding framework.

A Thematic Analysis was conducted to examine the experiences of the participants [22], using data management principles of the Framework Approach [23]. This encouraged breadth and depth in the exploration of the data by the research team whilst facilitating transparent and accessible data management and a clear audit trail. The analysis was conducted from a critical realist perspective; we sought the views and perceptions of participants in multiple contexts to expand our understanding of the reality of the topic at hand (e.g., [24]). Interviews were transcribed verbatim, read repeatedly to achieve familiarisation, and topics relevant to the research question were identified. Initial codes were generated from both groups’ data concurrently and collated into a single hierarchical coding framework of potential themes and sub-themes. Both explicit and latent themes were explored by the whole research team, thereby incorporating the different work and life experiences the team members brought to the study. Such experience included various levels of previous work with older adults, with physical activity, and with qualitative research. Additionally, the first author’s interest in this work was initially fuelled by her own experience of growing up, living, and working in low SES areas and driven forward by the powerful interactions with the participants themselves. In line with the Framework approach, matrices were produced in Excel to facilitate thematic and case-based analysis, with themes discussed and merged or split as necessary. The matrices assisted the analysis through identification of patterns within the data, identifying links between same or different phenomena and any notable absence of such links [25]. Final themes were elicited from analysis of such patterns and the interconnected nature of the themes (see Figure 1 for a thematic overview). Concurrent analysis allowed for a deeper exploration of each theme from a multi-perspective stance and allowed cross-case analysis to also highlight differences and similarities in perspectives.

## 3. Results

Table 1 below shows sociodemographic data for all participants. Of the 19 older adult participants, four identified as White Irish, one as British Pakistani, and 14 as White British. Two married couples participated and were interviewed individually. Ten older adults lived alone, 7 lived with their spouse, and two lived with other family members. Four participants across three households had access to a car. Participants were categorized as being highly active (*n =* 6), active (*n =* 3), somewhat active (*n =* 3), or low activity (*n =* 7) based on self-reported physical activity.

Of the eight trainer/provider participants, one was a volunteer, and the rest held paid positions. Most trainers/providers (*n* = 6) identified as White British (detailed breakdown not provided to protect participant identity due to the relatively small participant pool). Length of time working in physical activity for older adults ranged from 0.5 to 15 years (M = 5.64, SD = 4.73). The physical activities they offered included walking, Tai Chi, circuit training, dancing, walking football, and seated exercises.

The thematic analysis produced eight themes: being valued, tackling disadvantage, flexibility, familiarity, enjoyment, identity, group cohesion, and multi-functionality. A further theme explaining differences in perception of time in older age was found in the older adult sample alone and is discussed in detail in a separate publication [26]. Pseudonyms have been used for all quotes to preserve anonymity of participants. Gender, age, and activity levels are noted for older adults, and gender, age, and role are noted for trainer/providers.

### 3.1. Being Valued

A general lack of value of older adults in society was perceived:

*This world isn’t built for old people […] nobody respects you […] You’re given no respect for what you’ve done. You know, it’s… I don’t know. I think it’s very sad because we’ve got a lot that we could share with young people. But it’s got to be a two-way thing. You know, they’ve got to build us in when, you know, these people with budgets. They’ve got to build us into that as well. You know, we’ve got to be brought into the equation and then we’ve got a lot to share.*

(Linda, F, 68, Low activity)

This lack of value seemed exacerbated in low SES areas, where physical activities were often provided in shared community facilities rather than the dedicated spaces available in higher SES areas (e.g., tennis/golf clubs). There was a perception that older adults’ low-revenue activities were more prone to cancellation than others in shared facilities: *“Every time that the pool is needed, it’s always the [older adults’ aquafit] that get told ‘Your exercise is cancelled’”* (Kevin, M, 71, Highly active). Some trainer/providers tried to show older adults that they were valued: *“Talk to them, ask them how their day’s been. Ask them where they’re going after this. […] Listen to what they’ve got to say”* (James, M, 30, Trainer). Being offered something perceived as being desirable to others, knowledge of substantial discounts, or brand recognition increased acceptability:

*Zumba’s really popular, and that’s popular because it’s quite expensive normally to pay for if you were just to go into a gym or whatever. So, when we put those on locally, they’re received well. Cos it’s something that they have heard about.*

(Emma, F, 33, Provider)

Lack of funding led to lack of marketing, resulting in some older adults perceiving being overlooked in favour of other groups: *“I’ve never seen an advert about senior citizens swimming—there’s plenty of things about, you know, youngsters, school children, that sort of thing”* (Pam, F, 72, Low activity). Some trainers/providers understood this and felt a media campaign normalising physical activity in older age would show they were valued, e.g., similar to the “This Girl Can” campaign [27]: *“If they can do that for girls, why can’t they do it for older people”* (Jill, F, 40, Trainer).

### 3.2. Tackling Disadvantage

Older adults in low SES areas felt a strong sense of disadvantage, perceiving that those in higher SES areas received more acceptable and desirable physical activities:

*They won’t bring [Tai Chi] up here. They won’t think of [people from local area] doing things like that! […] they’re the ones [in wealthier area] that’ll get it. We wouldn’t get it, you know […] there’s not one place doing Tai Chi [in local area], there’s about three places [in wealthier area] doing it.*

(Linda, F, 68, Low activity)

Funding cuts in low SES areas had resulted in the loss of local facilities: *“We do miss our swimming pool”* (Sally, F, 78, Highly active); lack of services: *“There’s nowhere round here to dance”* (Jo, F, 69, Low activity)*;* and fear of loss of existing services: *“I’m trying to envisage what they, how they would access things and how they would go about it once [we] aren’t there”* (Emma, F, 33, Provider).

Lack of individual resources seemed to impact attending physical activity sessions in low SES areas. Available transport was seen as unfit for purpose, with older adults feeling they needed to leave classes early to ensure they did not miss transport and consequently feeling vulnerable in unsafe neighbourhoods: *“[They] started coming when they wanted, so I was missing half the [class…] because I’d have to go outside and wait. And you don’t want to be stood up there outside anything”* (Linda, F, 68, Low activity). Those with access to private transport did not have such issues with travelling to evening activities within low SES areas: *“Well not for where we would be going, because we’d only be going local, and I’d be driving[…] door to door, yeah”* (Diana, F, 71, Highly active).

Shared community resources were lost to other services, such as socialising spaces becoming offices: *“They used to go upstairs and have tea and biscuits […] you know what it’s like, funding”* (Jill, F, 40, Trainer). Trainers/providers found ways around their lack of resources with innovative use of existing services, e.g., ending a walk at a free coffee morning. However, the seeming inevitability of loss of services was also a focus within some delivery in an attempt to prepare older adults in low SES areas to cope when such loss occurred: *“Over the years, I’ve educated them. ‘So, if I can’t make it. If this building has to close […] you know at home that you can do this’”* (James, M, 30, Trainer).

Replacing community venues with centralised facilities outside the area did not seem to be acceptable: *“Merge them and make something bigger, and then you can put more funding into that and make it more successful. But it just doesn’t work”* (Emma, F, 33, Provider). Planners of such centralisation appeared unaware of the disadvantages that a lack of personal resources could have on older adults’ ability to attend a centralised facility: *“To the leisure centre, their thing was ‘Oh it’s only down the road.’ And it is only down the road, but not to maybe an older person who maybe doesn’t travel, or has walked there, or isn’t confident crossing main roads”* (Katie, F, 32, Provider).

### 3.3. Flexibility

Some providers mentioned that older adults seemed to lack flexibility around timetabling: *“If we have to change the instructor, or the time and the day, it kicks up such a fuss and we have to be very sympathetic to that”* (Katie, F, 32, Provider). Flexibility in older adults’ thinking around changing plans or incorporating physical activity into their routine was in fact evident, particularly through first-hand experience: *“But now I see that I can be active”* (Al, M, 77, Highly active). However, flexibility could be disrupted when higher priority conflicts occurred, such as family commitments: *“I used to go walking, but it was a funny time. That was on a Thursday. My daughter comes on a Thursday”* (Julie, F, 72, Highly active). Some whose working life had consisted of hard physical jobs also seemed to struggle with now viewing physical activity as a leisure pursuit to be engaged in during their traditional leisure time of the weekend: *“I said, “Not Saturday! [when asked to go on a walk]”* (Kath, F, 77, Somewhat active).

### 3.4. Familiarity

Reframing unfamiliar movements with familiar terms improved acceptability of the physical activity itself:

*They couldn’t remember the names of the movements, so they made up their own names and they were things like, “pulling the beer pump” or “changing the baby’s nappy,” or “washing the car,” but it made them, it helped them understand the [Tai Chi] movements, and as soon as that clicked in, that they renamed them, they could do the movements really comfortably.*

(Phil, M, 46, Trainer)

Familiarity with trainers helped to build trust, and there was a keen sense of loss when familiar trainers left: *“It was really a smack in the teeth when she went”* (Linda, F, 68, Low activity). Social contact with familiar others seemed to be a primary driver for engaging in physical activity: *“Knowing people who go to it, that’s the main thing”* (Sara, F, 74, Somewhat active). Fear of rejection in unfamiliar places adversely affected acceptability: *“If I sat there for an hour on my own and nobody came near me […] I just couldn’t cope with that. So that’s why I don’t go”* (Jo, F, 69, Low activity). However, some providers perceived a reluctance to travel beyond their familiar council area (ward) as something ingrained within the low SES community itself:

*You find that they will go to something that’s very, very near to them. But if you put the same class that they wanted on and you moved it to a different ward, they wouldn’t want to travel. And that’s not always easy to put something on in every ward. So, you will get that […] where they just won’t cross over […] I don’t know whether it’s because they feel safe in their ward or if… they’re being a traitor?*

(Emma, F, 33, Provider)

### 3.5. Enjoyment

Providing and promoting opportunities for enjoyment was seen as key to acceptability:

*Brand them as something else. Or put another spin on it, so people engage because it’s social, it’s fun, it’s friends. The health, the physical activity and everything else is a by-product. That’s something that might be our aim, but that’s not how we sell it.*

(Frank, M, 44, Trainer/Provider)

Life was not to be wasted on unenjoyable activities: *“You’ve got to enjoy what you’re doing, or otherwise don’t do it”* (Sally, F, 78, Highly active). When a regular physical activity was skipped, its absence was felt in both a lack of intrinsic enjoyment and also the lack of positive side effects usually experienced: *“You don’t have the same energy I don’t think […] If I don’t go [swimming], I do miss going”* (Shirley, F, 70, Active). Being immersed in physical activity helped some older adults to stay focused and enjoy living in the moment: *“It’s fantastic… I’m living in the present moment”* (Al, M, 77, Highly active).

The anticipation of seeing friends and socializing within and around classes was integral: *“We enjoy one another’s company while we’re doing it, so that’s the joyful part of it”* (Kevin, M, 71, Highly active); such social enjoyment could even help them to overlook physical ailments: *“You forget what’s wrong with you when you’ve got a crowd of people”* (Grace, F, 94, Low activity). Anticipated enjoyment of an activity could also help some older adults to overcome environmental barriers in their low SES neighbourhood:

*They were selling drugs down there […] and [my friend] said, “I’m not going down there anymore.” So, I started going, I got on my bike and went down on the bike on my own, yeah, and didn’t mind cos I was enjoying it.*

(Mo, F, 89, Low activity)

For some older adults in low SES areas physical activity seemed to be a way to enjoy a simple freedom: *“It [cycling] feels like freedom to me, you know”* (Diana, F, 71, Highly active); something they felt they had not had much experience of in their working life and were now being afforded through physical activities: *“We were allowed to be ourselves. We were given permission to have fun”* (Olive, F, 70, Highly active).

### 3.6. Identity

How older adults in low SES areas identified with certain physical activities appeared to be important, e.g., one participant indicated that they disliked walking as an activity but were willing to join walks with a purpose (e.g., history, nature), as this participant noted about dancing: *“I’ve never been a dancer in my life […] It’s not me. I don’t want to do it”;* but speaking on belly dancing: *“So I went, and it was a good laugh”* (Linda, F, 68, Low activity).

Identifying as physically active for some was related to hard physical jobs they no longer felt capable of: *“I think I’m past it”* (Susan, F, 80, Low activity). Some did not equate structured formal physical activity or exercise as something they would do in older age: *“Those days are over I think”* (Sam, M, 67, Active), preferring activities such as walking or gardening. For some, their sense of identity prevented involvement with activities provided by older-adult-based services: *“I don’t FEEL like a pensioner […] it’s just not for me”* (Olive, F, 70, Highly active). Others still very much identified as active people, but on further investigation, they were referring to a sense of busyness in their schedule rather than being overly physically active: *“I don’t think there’s anything more that I could really do, if I think about it”* (Kath, F, 77, Somewhat active).

### 3.7. Group Cohesion

Being part of a cohesive group was important for maintaining physical activity: *“When you’re with people that you know, you’re more encouraged to go, aren’t’ you? […] because if you’re on your own and you think, ‘Oh I won’t bother, I’ll leave it’”* (Claire, F, 67, Somewhat active). Providers spoke of the process of a room full of strangers becoming a cohesive group:

*That’s a really lovely thing to see, when you start a new group, and you’ve got all these strangers around the room, and they kind of don’t know each other, or some of them will know each other, and they don’t know what they’re doing, they don’t know what’s expected. And then over a period of time, you see it, something happens, a kind of, it settles, you know? It’s like a cake in the oven, you know, isn’t it? [laughing] You put all the ingredients together, and then magically it turns into a cake.*

(Fiona, F, 61, Trainer/Provider)

Group cohesion needed managing to incorporate new members: *“It does stop other people, if somebody doesn’t make you welcome, you stop the class from growing”* (Sara, F, 74, Somewhat active).

### 3.8. Multi-Functionality

Attendance at physical activity sessions addressed multi-functional needs for older adults including social: *“Well, 50 per cent is activity, and 50 per cent is sociability with the people”* (Sara, F, 74, Somewhat active); or leisure interests: *“We [the walking group] go to the science museum and the other museums, you know, and I like that kind of thing […] You’re absorbing knowledge as well, you see, and I find that interesting”* (Liz, F, 74, Active). For others, solitary physical activity was acceptable only if it also addressed other needs: *“I’d walk up to the shops on my own alright but not to go out for a walk on my own”* (Mo, F, 89, Low activity). Multi-functionality sometimes hindered the intensity of physical activity, as noted by both trainers/providers and some more active older adults: *“We struggle to maintain a good walking pace when the people that walk with me are often interested in nature and stopping to observe things”* (Mary, F, 62, Trainer/Provider); *“It drags me down when you’re walking slow, waiting for people or walking in a group that’s not walking, if you know what I mean […] I want the walk”* (Diana, F, 71, Highly active). However, maintenance was encouraged due to multi-functionality, e.g., walking to and from a sociable physical activity to meet social needs despite weather stopping much of the session activity, ensuring that the routine of attending was maintained and a couple of short walks still undertaken:

*If it was pouring down with rain we wouldn’t say “Oh, we won’t bother going to the club” you know, we’d still [walk] round. I mean if it’s gardening, we wouldn’t go out in the garden. We’d just sit there and have a cup of tea and a chat, you know, and then come home.*

(Ben, M, 74, Low activity)

Lack of multi-functionality coupled with limited resources could result in older adults in low SES areas pitting physical activity against other activities: *“…more to do with how that encroaches in my life balance of how much time I want to spend doing that, as against something else that I want to do”* (Sam, M, 67, Active).

## 4. Discussion

This study explored individual perspectives on older adults’ acceptability of physical activity provision from both older adults living in and trainers/providers working in low SES areas. The eight themes produced together show the varied and complex issues related to provision and engagement with physical activity for older adults in lower SES areas. The older adults often felt disadvantaged and undervalued when comparing themselves to older adults in higher SES areas and to younger people in general. Their sense of value increased when they felt that their needs were taken into consideration and when they were provided with physical activities deemed appealing to others.

Several studies have shown that reduced amenities and limited resources in low SES areas negatively impact engagement in leisure-time physical activity [6,7,8,28]. In the present study, trainers/providers working within low SES areas generally understood the negative impact limited resources had on older adults’ ability to engage in physical activity and did their utmost to make the older adults who attended feel valued by listening and providing time for them all to talk, thereby providing social contact and promoting group cohesion. However, they often felt powerless in the face of the apparent lack of such understanding by facility management, who further compounded the disadvantages faced by older adults living in lower SES areas by centralising physical activity services, cancelling classes with little notice, and removing provision in low SES areas.

The feelings regarding inequality of provision in the current study are perhaps unsurprising, as it has been shown that parity of provision is often absent in low SES areas. A study of the spatial distribution of facilities [29] found low/medium SES areas contained fewer physical activity facilities overall and fewer free facilities than higher SES areas. A Spanish study [30] found such a lack of local convenient facilities negatively impacted the physical activity levels of older women (but not older men or younger adults) in lower SES areas. The lack of societal value older adults in lower SES areas experienced in the current study was stark when their classes were the first to be cancelled, when current provision was taken away, and when attractive provision was offered in more affluent neighbourhoods by the same provider but not in their neighbourhoods. Furthermore, the removal of vital social facilities within municipal buildings in low SES areas further illustrated the seeming lack of understanding of older adults’ needs by facility management. To feel disregarded in such a manner was no inducement to engage in physical activity even though some trainers/providers tried to equip older adults with the knowledge to continue independently should local provision close. Trainers/providers also tried to tackle lack of social facilities as best they could with innovative use of existing free services, but such opportunities were rarely available. Furthermore, the removal of opportunities to socialise around physical activity sessions in the lower SES area facilities added further to the inequality by reducing the multi-functionality of the event, where older adults with limited resources may be forced to choose between attending a socially or physically beneficial activity.

The issue of these limited resources needs to be addressed in order to tackle such disadvantage. Lack of access to private or public transport, together with centralised services moved further afield, requires greater expenditure of both time and energy merely to attend a physical activity session, making engagement challenging for those with limited resources [26]. Furthermore, as noted in the current study, the impact of unreliable transport was compounded by feelings of vulnerability in unsafe neighbourhoods, again reducing the acceptability of attending physical activity sessions for many. It should be noted that, even though all participants lived in lower SES areas, actual level of income was not recorded in the current study. Given this, some participants may have had greater funds that increased their possible access to private transportation and other amenities further afield. The relationship between neighbourhood safety and leisure-time physical activity has been reported as a barrier to physical activity for older adults in low SES areas [9]. Some older adults in the current study suggested that the pleasure they derived from sessions was enough for them to overcome such environmental barriers but not for others they knew.

The perceived added value of group activities for lifestyle behaviour change has been noted as more important for low SES older adults than those of higher SES [28]. The current study suggests that facilitating strong social bonds and group cohesion helped older adults to maintain regular physical activity, perhaps due to a perceived obligation to group members [28]. Promotion of the social aspect of group physical activities may help older adults in low SES areas to identify more with the pleasure of leisure-time physical activity rather than to see it as hard work, something that many have had enough of. Although we did not collect data on how many physical activity classes or other social classes participants in the current study attended per week, such information may illustrate further the preferences for engaging socially or not within this target population. Social physical activity sessions did seem highly acceptable in low SES areas across the majority of participants regardless of their activity levels. This suggests that more sociable provision may encourage more older adults to leave their house, which is itself positively associated with higher levels of physical activity [7]. Focusing on social aspects rather than physical aspects of provision was also seen to be more acceptable to older adults in low SES areas by the trainers/providers who worked there, both in practice and in marketing. Such findings are in line with social goals being more relevant motivators to being physically active for older adults than younger adults [31].

### 4.1. Strengths and Limitations of Study

The multi-perspective aspect of this study gives insight into issues of acceptability of physical activity provision from both older adults and trainers/providers delivering physical activity in lower SES areas. This approach allowed us to see different facets of the same issue in an understudied context, which could inform future provision. The study was conducted in a city that is ranked third highest for deprivation in England [20] and recruited in areas with higher deprivation for older adults within that city. This use of an objective measure of SES for recruitment area took into account salient environmental and household issues specific to the study population, without experiencing possible underreporting of sensitive information, as can occur with individual measures of SES [32]. However, there was a broad range of education among older adult participants, with the majority having secondary school education or lower but two participants having PhDs. Collecting household income data may have illustrated differences in individual circumstances.

Older adults’ activity levels used to describe the sample were defined in accordance with recommended guidelines; however, their self-report has limitations of lower accuracy compared to objective measures [33]. Although purposive sampling resulted in wide range of activity levels within older adults and a wide range of work sectors within trainers/providers, there was low gender and ethnic diversity. These latter two factors somewhat limit the insight gained, as does the focus on urban areas.

### 4.2. Implications

These findings suggest that providing multi-functional desirable physical activities that focus on fun, social, or leisure interests may allow older adults with limited resources to be physically active without sacrificing other desired activities. This approach may also capture more inactive older adults simply looking for fun, social activities who would not necessarily be drawn to a purely physical activity but who may nevertheless experience the health/wellbeing benefits as a by-product. Consistent, familiar, local provision may reduce expenditure of financial, physical, and mental resources whilst retaining social networks and encouraging maintenance of physical activity. Further research is required to confirm and quantify these findings in a larger, more diverse sample.

## 5. Conclusions

The present research sets out multiple facets of physical activity provision that are linked to acceptability of physical activity to older adults in low SES areas. To increase acceptability of physical activity for older adults in low SES areas, providers should address the lack of personal perceived value felt by many older adults. Equitable provision of physical activities addressing multiple needs (e.g., social, hobbies) may allow older adults with fewer resources to be physically active without sacrificing other needs. Such provision needs to be social, familiar, and enjoyable, so it may be perceived as a leisure-time activity. Facilitation of social interaction creates strong social bonds, potentially fostering maintenance of physical activities. Addressing these issues is likely to produce greater acceptability and thereby greater engagement in physical activity in this population.

## Figures and Tables

**Table 1:** Sociodemographic Characteristics of Participants.

Sociodemographic Characteristic	Older Adults		Trainers/Providers	
	*n* = 19 Age in Years 67–94 (M = 74.4, SD = 7.1)		*n* = 8 Age in Years 30–62 (M = 43.5, SD = 12.5)	
	*n*	%	*n*	%
Gender				
Female	15	79	5	62.5
Male	4	21	3	37.5
Marital Status			Not reported	Not reported
Married	7	36.8		
Widowed	6	31.6		
Divorced	3	15.8		
Single	3	15.8		
Education			Not reported	Not reported
Did not complete secondary education	5	26.3		
Completed secondary education	7	36.8		
Further education	5	26.3		
PhD	2	10.5		

## Data Availability

The data that support the findings of this study are available on reasonable request from the corresponding author. The data are not publicly available due to privacy or ethical restrictions.

## Supplementary Materials

The following are available online at https://www.mdpi.com/article/10.3390/ijerph182211784/s1. Supplementary S1: Older Adults’ Interview Schedule; Supplementary S2: Trainers/Providers’ Interview Schedule
