# Supplementary material for: The Acceptability of Physical Activity to Older Adults Living in Lower Socioeconomic Status Areas: A Multi-Perspective Study
Source: Int J Environ Res Public Health. 2021 Nov 10;18(22):11784. doi: 10.3390/ijerph182211784 (PMC8619977; doi:10.3390/ijerph182211784)
Supplement: Supplementary file 1 [file ijerph-18-11784-s001.zip › Supplementary S1 & S2 Interview Schedules IJERPH 300921.pdf]

Supplementary S1

Interview Schedule Older Adults

Older Adults and Acceptability of Physical Activity

Opening:

So I'll be asking some questions about your experiences of physical activity and how you feel about it, what's acceptable to you and what's not. There are no right or wrong answers, I just want to hear your views. You can take a break at any time and you don't have to answer a question if you'd rather not. Is that okay?

1. **Say you went to the doctors and your GP suggested that you increase your 'physical activity' levels, what would you think he or she was encouraging you to do?**

2. **How would you describe yourself in terms of physical activity levels?**
   (Explore in depth)
   If active – what motivates you to be so active?
   If not – what stops you from being more active?
   If PA level lower than recommended: What would need to change for you to increase your level of physical activity?

3. **How do you feel about physical activity?**
   (Prompt on different types of activity – dancing, walking, exercise classes, gardening)

   If enjoy: What kinds of physical activity do(did) you enjoy/enjoy the most? *(If references made to previous enjoyment but not present enjoyment, probe further to find out what has changed to stop them enjoying it now – what could help them to enjoy it again).*

   If don't enjoy PA: What is it about physical activity that you don't like? Get specific.

   What **benefits** do you think being physically active has for you? **OR**
   What benefits do you think there would be from being more physically active?
   (Explore physical/cognitive protection and mental wellbeing, social interaction, functionality, family benefits)

   Is there anything that would **worry** you about **increasing** your level of physical activity? What would **ease those worries**?

4. **What are the range of activities like in your area? Are there any physical activities that you would like to do, but do not have access to?**
   What would that be like, when, how many times a week, where? How would it be delivered? What would the trainer/leader be like? Who would you go with?

5. **Do you have any inactive friends/family/neighbours? What do you think about their levels of activity? What do you think could help them to get active?**

6. **How do you think physical activity has/can become an acceptable part of your everyday life?**

7. **Are there any groups of people who you think would benefit from being more physically active? What do you think could be done to help them or motivate them to be more physically active?**

8. **What makes it easier/harder for you personally to be physically active?**

9. **Has any health professional ever suggested that you become more physically active?**
   Yes: How did you feel about that? What was the effect? Why? What could they have done differently?
   No: How would you feel if one did?

10. **Is there anything else you would like to add?**

Supplementary S2

Interview Schedule Trainers/Providers

Older Adults and Acceptability of Physical Activity

First I'll ask a couple of background questions on your involvement with older adults and physical activity and then I'll focus more on your experiences of this work. There are no right or wrong answers, I just want to hear your views. You can take a break at any time and you don't have to answer any question you'd rather not. Is that okay?

1. **How long have you been involved in providing/delivering physical activity to older adults?**

2. **Is there a reason you became involved with physical activity for older adults?**

3. **What sorts of physical activity do you think older adults like to do? What is it about these activities that you think appeals to them?**

4. **What do you think the provision of physical activities for older adults is like in more deprived areas?**

5. **What physical activities do you provide/deliver?**
   Do you offer different types of physical activities or deliver them in different ways for different populations? Is it for a set age range and above or is it based more on ability?

6. **What feedback have you received about the physical activities you provide?**

7. **How well attended are the physical activities that you provide? Has this changed over time?**

8. **Are you trying to increase older adult engagement in physical activity?**
   If 'Yes' – How are you going about this?
   If 'No' – Can you think of anything that could be done to increase older adults attending your classes/centres or engaging in physical activity in general?

9. **Do you see any barriers to older adults in your area engaging in the activities you already provide/deliver?**
   Follow up: have you had any feedback from older adults about barriers – perhaps why they won't be coming anymore?

10. **Is there anything else you would like to add?**
